# *CHL1* gene acts as a tumor suppressor in human neuroblastoma

**DOI:** 10.18632/oncotarget.25403

**Published:** 2018-05-25

**Authors:** Marzia Ognibene, Gabriella Pagnan, Danilo Marimpietri, Davide Cangelosi, Michele Cilli, Maria Chiara Benedetti, Renata Boldrini, Alberto Garaventa, Francesco Frassoni, Alessandra Eva, Luigi Varesio, Vito Pistoia, Annalisa Pezzolo

**Affiliations:** ^1^ Laboratorio Cellule Staminali Post Natali e Terapie Cellulari, Istituto Giannina Gaslini, Genova, Italy; ^2^ Laboratorio di Oncologia, Istituto Giannina Gaslini, Genova, Italy; ^3^ Laboratorio di Biologia Molecolare, Istituto Giannina Gaslini, Genova, Italy; ^4^ Animal Model Facility, IRCCS Azienda Ospedaliera Universitaria San Martino, IST, Istituto per la Ricerca sul Cancro, Genova, Italy; ^5^ Laboratorio di Anatomia Patologica, Ospedale Pediatrico Bambino Gesù, Roma, Italy; ^6^ Area di Immunologia, Ospedale Pediatrico Bambino Gesù, Roma, Italy; ^7^ Divisione di Oncologia, Istituto Giannina Gaslini, Genova, Italy; ^8^ Present address: Laboratorio di Immunologia Clinica e Sperimentale, Istituto Giannina Gaslini, Genova, Italy

**Keywords:** neuroblastoma, CHL1 gene, differentiation, apoptosis, autophagy

## Abstract

Neuroblastoma is an aggressive, relapse-prone childhood tumor of the sympathetic nervous system that accounts for 15% of pediatric cancer deaths. A distal portion of human chromosome 3p is often deleted in neuroblastoma, this region may contain one or more putative tumor suppressor genes. A 2.54 Mb region at 3p26.3 encompassing the smallest region of deletion pinpointed *CHL1* gene, the locus for neuronal cell adhesion molecule close homolog of L1. We found that low *CHL1* expression predicted poor outcome in neuroblastoma patients. Here we have used two inducible cell models to analyze the impact of CHL1 on neuroblastoma biology. Over-expression of *CHL1* induced neurite-like outgrowth and markers of neuronal differentiation in neuroblastoma cells, halted tumor progression, inhibited anchorage-independent colony formation, and suppressed the growth of human tumor xenografts. Conversely, knock-down of CHL1 induced neurite retraction and activation of Rho GTPases, enhanced cell proliferation and migration, triggered colony formation and anchorage-independent growth, accelerated growth in orthotopic xenografts mouse model. Our findings demonstrate unambiguously that CHL1 acts as a regulator of proliferation and differentiation of neuroblastoma cells through inhibition of the MAPKs and Akt pathways. *CHL1* is a novel candidate tumor suppressor in neuroblastoma, and its associated pathways may represent a promising target for future therapeutic interventions.

## INTRODUCTION

Neuroblastoma (NB) is a complex disease and many factors, such as age at diagnosis, stage, as well as pathologic and genetic features of the tumor, determine whether it will spontaneously regress or metastasize and become refractory to therapy [1]. NB originates from primordial neural crest cells that subsequently develop into sympathetic ganglia and the adrenal medulla. NB is heterogeneous in clinical presentation, course, and overall prognosis, ranging from infants with tumors that can spontaneously regress; to children who have localized tumors with favorable genomic characteristics and excellent overall survival following cytotoxic chemotherapy; to critically ill older children, adolescents, or young adults with widely disseminated disease growing relentlessly despite intensive multimodal chemo-radiotherapy [2].

We have recently reported a sub-microscopic constitutional deletion of the short arm of chromosome 3, extended to band p26.3 and encompassing 2.54 Mb in size, in a 17-years-old male with localized NB [3]. The deletion breakpoint disrupted the *CNTN4* gene, and the neighboring *CNTN6* and *CHL1* genes were hemizygously deleted. These three genes encode neuronal cell adhesion molecules [3]. Further, 3p deletion is an independent predictor of NB progression [4], lending support to the assumption that distal 3p harbors genetic information mediating tumor suppression [5]. Studies aimed at identifying genes whose expression is consistently altered by chromosomal losses in 3p deleted tumors have allowed to define a 5.6 Mb region of common loss containing six down-regulated genes: *CHL1*, *CNTN4*, *CRBN*, *LRRN1*, *SETMAR* and *ARL8B* [6]. Loss-of-function mutations of *CHL1* have been reported in NB [7]. The protein encoded by *CHL1* is a member of the L1 family of neural cell adhesion molecules expressed in subpopulations of developing neurons in the central and peripheral nervous systems [8]. CHL1 expression persists at low levels in the mature brain in areas of high plasticity [8]. CHL1 plays important functional roles in the development and regeneration of the nervous system [8]. The *CHL1* gene is involved in general cognitive activities and some neurological diseases [9], and recent studies point to a role in neurite regeneration [10]. Of note, it has been proposed that defects in neuritogenesis regulating genes represent an important category of tumor-driving events in NB, and tumors with genomic defects in neuritogenesis genes cluster in high-risk NB [11]. CHL1 driven neuronal differentiation is mediated by the cytoskeleton. CHL1 interacts with and recruits to the cell surface membrane cytoskeleton-linker proteins such as ankyrin, the ezrin-radixin-moesin family, and βII spectrin [12, 13]. Mice deficient in the orthologous gene *Chl1* display misguided axons within the hippocampus and olfactory tract, and anomalies in behavior [14]. In addition, deletion of one copy of *CHL1* gene might be responsible for mental defects in patients with 3p deletion syndrome [15].

Several reports suggest that *CHL1* is involved in carcinogenesis [16, 17]. *CHL1* was designated as a candidate tumor suppressor gene in uveal melanomas based on the decreased expression in samples from patients with grim clinical outcome [18]. Furthermore, ectopic expression of CHL1 in nasopharyngeal carcinoma cells inhibited their clonogenicity and migration as compared with parental cells without CHL1 expression [19]. The present study was undertaken to discover the molecular mechanisms regulated by CHL1 in NB.

## RESULTS

### Decreased *CHL1* expression is significantly associated with poor prognosis in neuroblastoma

We analyzed the gene expression of 174 primary NB samples profiled by the Affymetrix HG-U133plus2.0 platform to identify groups of patients with different CHL1 expression. We selected a threshold value to determine the expression level (low or high) of CHL1 using the Elbow method. The threshold value divided the dataset in two groups: a group with very low CHL1 expression 133/174 tumors (76.4%), and a group with mean to high expression 41/174 tumors (23.6%). To study the expression of CHL1 in the presence of the 3p deletion we have identified in the dataset nine samples carrying 3p deletion containing *CHL1* gene. All 3p-deleted tumors showed low CHL1 expression. This result indicated that 3p deletion induced a reduction of *CHL1* gene expression.

**Figure 1:**
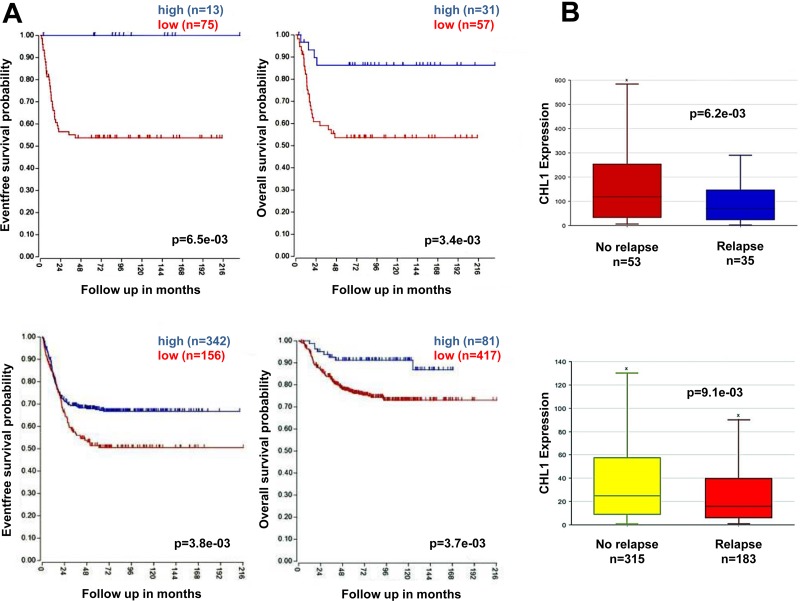
Low CHL1 expression correlates with poor prognosis in NB patients (**A**) Using the neuroblastoma Versteeg (top) and SEQC (bottom) patients data-sets in the R2 Genomics Analysis and Visualization Platform (http://r2.amc.nl), patients were divided into high (blue) and low (red) *CHL1* gene expression groups by median-centered Log2 ratios, and survival curves were generated. Event-free survival (bottom left) and overall survival (right) curves are shown together with patients numbers in parentheses. (**B**) Relative *CHL1* expression levels were plotted in patients with and without relapse from the Versteeg (top) and SEQC (bottom) patients data-sets. *n* = patients number.

Next, we evaluated the association of *CHL1* gene expression with NB patient outcomes, using online microarray data from two independent NB patients data-sets (Versteeg and SEQC) obtained from the R2 Genomics Analysis and Visualization Platform (http://r2.amc.nl). The resulting figures and *p* values were downloaded. The optimal cut-off for survival analyses was chosen as the expression value where the log-rank statistic for the separation of survival curves reached a maximum. Low expression of *CHL1* was significantly associated with reduced event-free survival and overall survival rates in two patient cohorts (Figure 1A). *CHL1* gene expression was significantly lower among patients who experienced disease relapse, compared to those who did not have disease relapse (Figure 1B).

### *CHL1* drives neuronal differentiation of neuroblastoma cells

**Figure 2:**
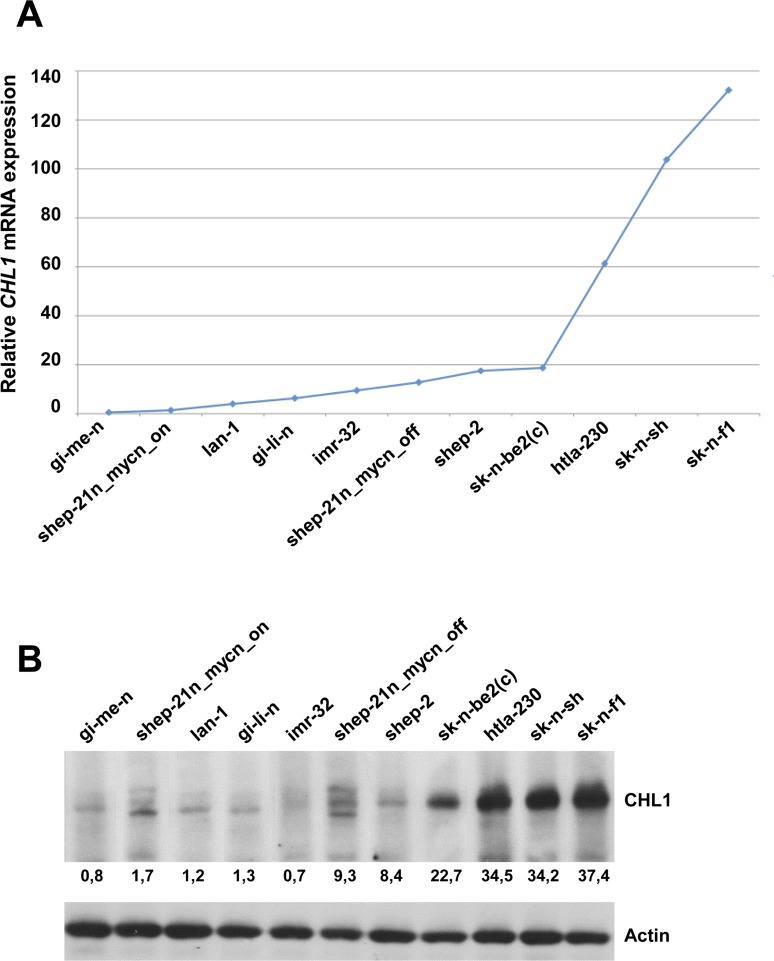
CHL1 expression in 11 NB cell lines (**A**) *CHL1* mRNA level, quantified by q-RT-PCR, was very low in GI-ME-N, SH-EP-21N_mycn_on, LA-N-1, GI-LI-N, IMR-32, SH-EP-21N_mycn_off, SH-EP-2, SK-N-BE2(C); mean-high in HTLA-230, SK-N-SH and SK-N-F1. (**B**) CHL1 protein levels analyzed by Western Blot in the same NB cell lines. Lower numbers indicate densitometric values.

First, a panel of 11 NB cell lines were screened for *CHL1* baseline expression by quantitative real-time PCR and Western blot analyses. As shown in Figure 2A and 2B, *CHL1* mRNA expression and the related protein level were very low in GI-ME-N, SH-EP-21N_mycn_on, LA-N-1, GI-LI-N, IMR-32, SH-EP-21N_mycn_off, SH-EP-2; and mean to high in SK-N-BE2(C), HTLA-230, SK-N-SH, SK-N-F1.

**Figure 3:**
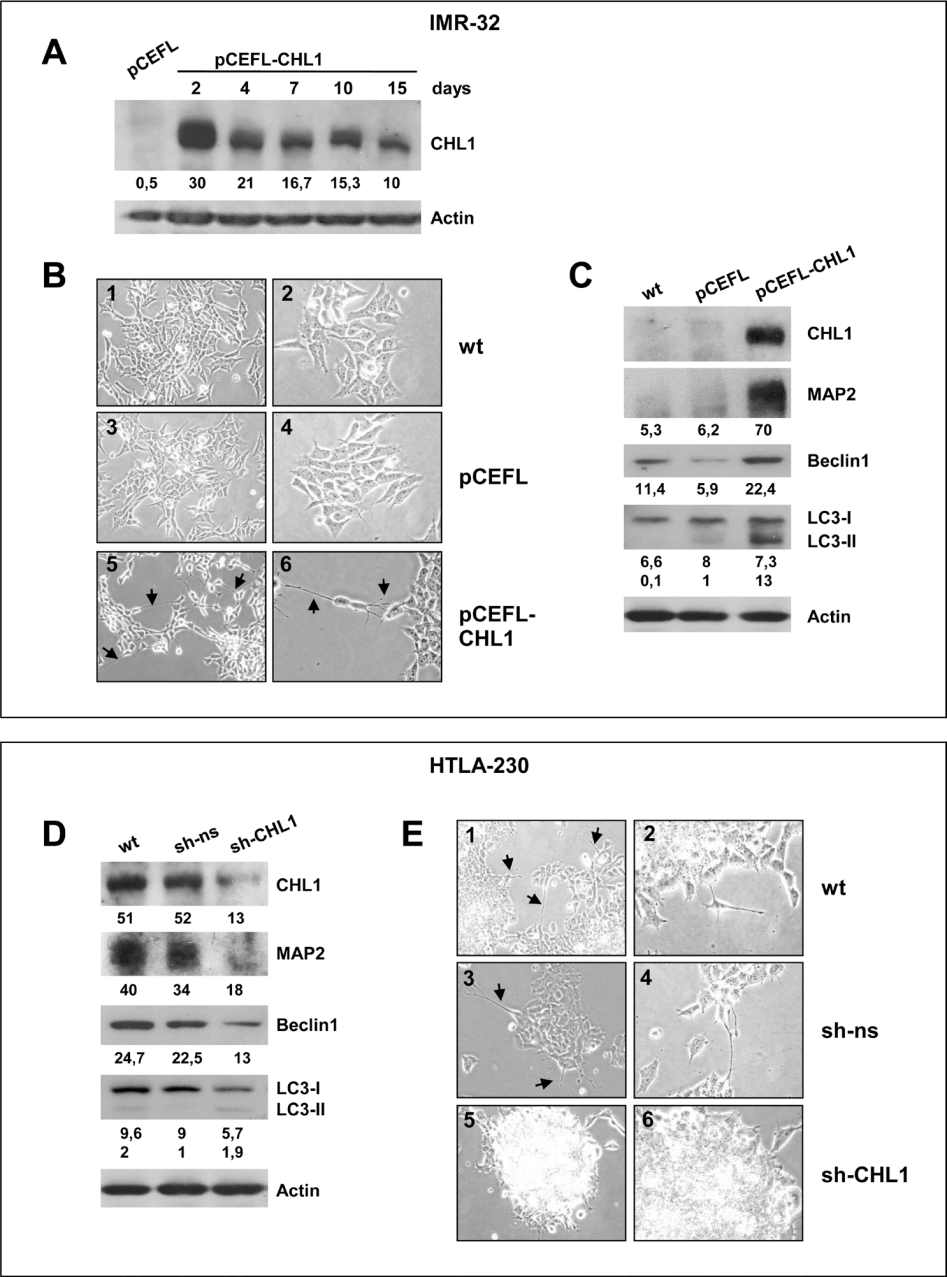
CHL1 and neuronal differentiation of NB cells (**A**) IMR-32 cells transiently transfected with pCEFL-CHL1 and lysed 2, 4, 7, 10 and 15 days after transfection were subjected to Western blot analysis and probed with anti-CHL1 antibody. The empty vector (pCEFL) was used as negative control. (**B**) Morphological characteristics of IMR-32 cells wild type (wt) (1, 2); or transfected with pCEFL (3, 4); or with pCEFL-CHL1 (5, 6). Arrows indicate the neurite-like extensions. (Magnification 20× left panels, 40× right panels). (**C**) Protein lysates from IMR-32 cells wt, or transfected with pCEFL, or with pCEFL-CHL1 were probed with anti-MAP2, anti-Beclin1, or anti-LC3 antibodies. (**D**) Protein lysates from HTLA-230 cells wt, or transfected with sh-ns or with sh-CHL1 were probed with anti-CHL1, anti-MAP2, anti-Beclin 1 and anti-LC3 antibodies. Lower numbers indicate densitometric values. (**E**) Morphological characteristics of HTLA-230 cells wt (1, 2), or stably transfected with non-silencing control shRNA (sh-ns) (3, 4) or with silencing CHL1 shRNA plasmid (sh-CHL1) (5, 6). Cells transfected with sh-CHL1 tend to grow in clusters and may form clumps of rounded cells on top of one another. Arrows indicate the neurite-like extensions detectable in cells wt and in controls (Magnification 20× left panels, 40× right panels).

We chose IMR-32 cell line for further experiments since it displayed very low CHL1 expression, and over-expressed MYCN that is known to increase NB malignant potential [1]. IMR-32 cell line was transiently transfected with the modified eukaryotic expression vector pCEFL-CHL1, or with the empty vector (pCEFL) as negative control. CHL1 expression was strongly increased in CHL1-transfected IMR-32 cells compared with control cells, and persisted at high level of expression until 15 days (Figure 3A). CHL1 over-expression induced changes in the morphology of IMR-32 cells that showed an higher number of neurite-like extensions respect to control cells (35 ± 9 *vs* 3 ± 1; *p* = 0.005) (Figure 3B). Accordingly, CHL1 over-expressing IMR-32 cells showed *de novo* expression of the neural differentiation marker MAP-2 (Microtubule Associated Protein-2) (Figure 3C), which interacts with microtubule filaments and plays a role in the initiation of neurite growth [20]. Our data show that the over-expression of CHL1 triggered autophagy, as evidenced by up-regulation of Beclin-1 and autophagic-vesicle-form LC3-II (Figure 3C).

Additionally, we examined the effects of CHL1 inhibition in HTLA-230 another MYCN over-expressing cell line that had mean to high CHL1 expression. We knocked down *CHL1* in HTLA-230 cells line through the stable transduction of a pool of three lentiviral small hairpin RNA (shRNA) plasmids (sh-CHL1), achieving 75% reduced expression (Figure 3D). CHL1 silencing decreased MAP-2 and autophagy markers Beclin1 and LC3-II expression (Figure 3D). HTLA-230 cells transfected with sh-CHL1 showed an undifferentiated morphology, and grew in clusters forming clumps of rounded cells on top of one another. These features were not detected in HTLA-230 cells wt or transfected with a non-silencing control shRNA plasmid (sh-ns), which grew in small aggregates and often showed neurite-like protrusions (Figure 3E). Moreover, low CHL1 expression induced a reduction of the number of neurite-like extensions respect to control cells (2 ± 4 *vs* 15 ± 2; *p* = 0.003), and a general lower differentiation degree.

### *CHL1* decreased neuroblastoma cell proliferation and increased apoptosis

**Figure 4:**
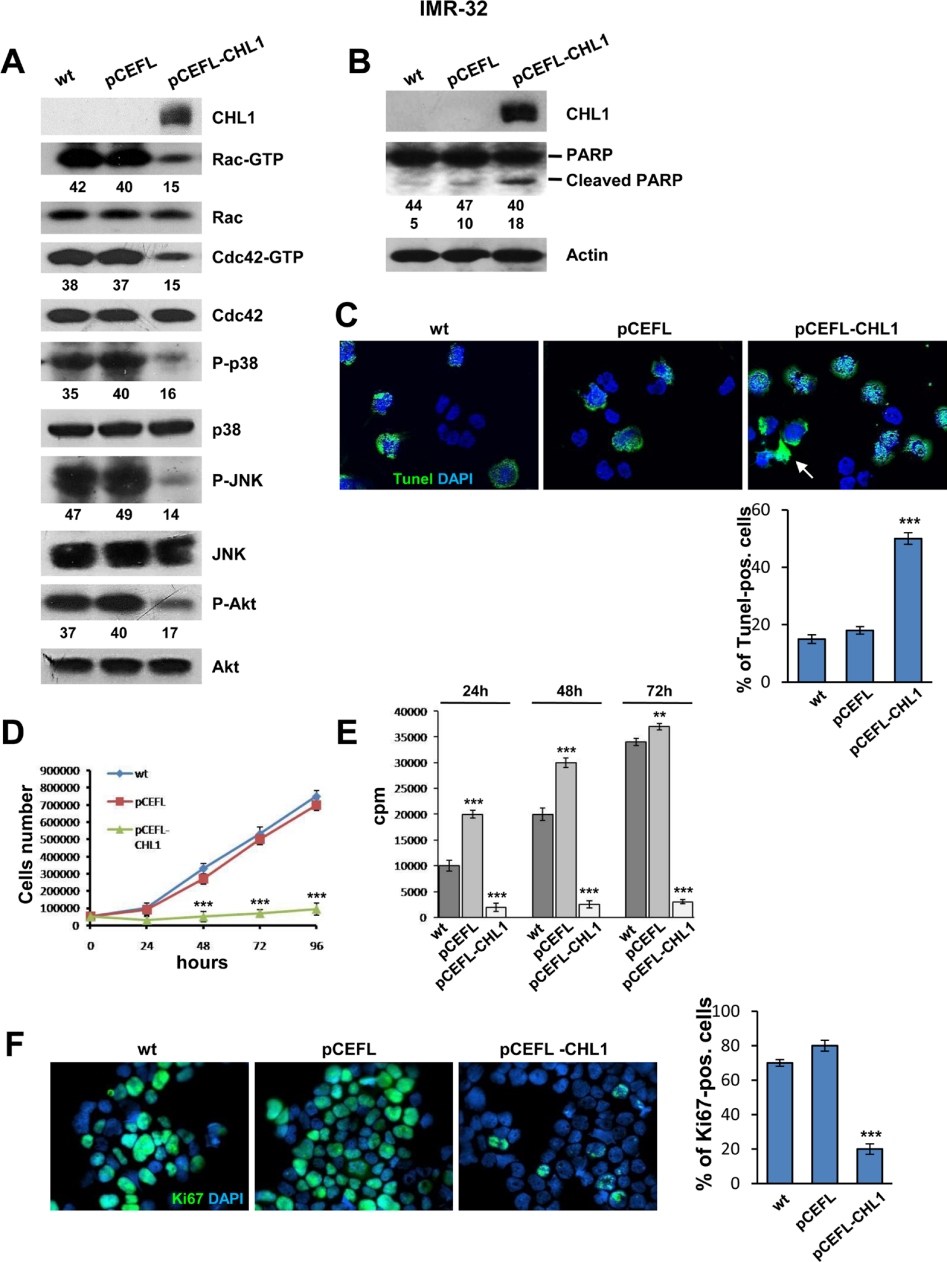
Over-expression of CHL1 inhibits proliferation of NB cells (**A**) Protein lysates from IMR-32 cells wt, or transfected with pCEFL or with pCEFL-CHL1 were collected and subjected to GST-PAK pull-down assay and anti-Rac or anti-Cdc42 Western blot analysis. The same protein lysates were blotted again and probed with anti-phospho-p38, anti-phospho-JNK, and anti-phospho-Akt antibodies. Lower numbers indicate densitometric values normalized to each total protein expression. (**B**) IMR-32 wild type or transfected with pCEFL or with pCEFL-CHL1 were subjected to Western blot and probed with anti-PARP antibody to test apoptosis. The level of apoptosis assessed by the cleaved-PARP was greater in IMR-32 cells transfected with pCEFL-CHL1 than in IMR-32 wt or transfected with pCEFL. (**C**) TUNEL-positive cells were examined by fluorescence microscopy (Magnification 60×). Staining with TUNEL (green) revealed chromatin condensation and the typical morphological changes characteristic of apoptosis (arrow). Quantitative analysis of apoptosis was carried out by counting TUNEL-positive and negative cells. Nuclei were counterstained with DAPI (blue) (Three independent experiments ± SD). (**D**) IMR-32 cells wt, or transfected with pCEFL or with pCEFL-CHL1 were plated in 12-well plastic plates and cultured for 4 days. Every day, cells were trypsinized and counted. Data are representative of three independent experiments ± S.D. (**E**) IMR-32 cells wt or transfected with pCEFL or with pCEFL-CHL1 were subjected to the [^3^H]thymidine incorporation assay. Cells were analyzed with a β-counter to quantify the amount of radioactivity incorporated after 18 hours of incubation (cpm = counts per minute). (Three independent experiments ± S.D). (**F**) Immunofluorescence analysis of proliferating cells using anti-Ki67 (green). Cells were counterstained with DAPI to visualize nuclei (blue). (Magnification 40×). (Three independent experiments ± SD).

CHL1 interacts with the p21-activated kinases (PAKs) [21] and mitogen-activated protein kinases (MAPKs) pathways [22]. Here we investigated if over-expression of CHL1 affected small GTPases activity and the consequent phosphorylation of specific MAPKs. CHL1 over-expression inhibited Rac and Cdc42 activation by approximately 60% in IMR-32 cells transfected with pCEFL-CHL1 compared to controls IMR-32 cells wt or transfected with pCEFL (Figure 4A). Next, we evaluated the influence of CHL1 over-expression on the activation of the molecular effectors of Rho GTPases, and specifically on the serine-threonine phosphorylation of p38 mitogen-activated protein kinase (p38) and Jun N-terminal kinase (JNK) [23]. As shown in Figure 4A, phosphorylation of both p38 and JNK was reduced in cells over-expressing CHL1 by approximately 50% and 70%, respectively.

To investigate the connection between CHL1 and apoptosis in NB cells, we evaluated the activation state of RAC-alpha serine-threonine-protein kinase (Akt), the major downstream effector of phosphoinositide 3-kinase (PI3K), in turn activated by Rac and Cdc42 GTPases, and strictly involved in cell growth and survival [24]. Phosphorylation of Akt (pAkt) prevents apoptosis and induces cell proliferation and transformation. pAkt hyper-activation contributes to many pathophysiological conditions, including human cancers, and is closely associated with poor prognosis and chemo- or radio-resistance [25]. Over-expression of CHL1 in IMR-32 cells induced a decrease of pAkt by approximately 58% in comparison to controls (Figure 4A). Western blot detection of the DNA repair enzyme Poly ADP-Ribose Polymerase (PARP) cleavage has been extensively used as an indicator of apoptosis [26]. Here we observed an enhanced expression of cleaved PARP in IMR-32 cells transfected with pCEFL-CHL1 when compared to control cells (Figure 4B). Next, we performed TUNEL-assay, which labels fragmented DNA as marker of programmed cell death. The proportion of TUNEL-positive cells was significantly higher in IMR-32 cells transfected with pCEFL-CHL1 (50%) than in IMR-32 cells wt (15%), or IMR-32 cells transfected with pCEFL (18%) (Figure 4C). The augmentation of TUNEL-positive cells, accompanied by increased expression of cleaved PARP in IMR-32 cells transfected with pCEFL-CHL1, suggests that over-expression of CHL1 induces apoptosis in NB cells through pAkt down-regulation. On the other hand, growth rate of CHL1 over-expressing IMR-32 cells was approximately eight fold lower than that of control cells, as assessed by cell count (Figure 4D). Accordingly, IMR-32 cells transfected with pCEFL-CHL1 underwent a significantly decreased cell proliferation compared with control cells as measured by ^3^H-thymidine incorporation (Figure 4E). Furthermore, staining of IMR-32 cells transfected with pCEFL-CHL1 with anti-Ki67 mAb, which identifies proliferating cells, showed that Ki67 positivity was significantly lower respect to control and wt cells (Figure 4F).

**Figure 5:**
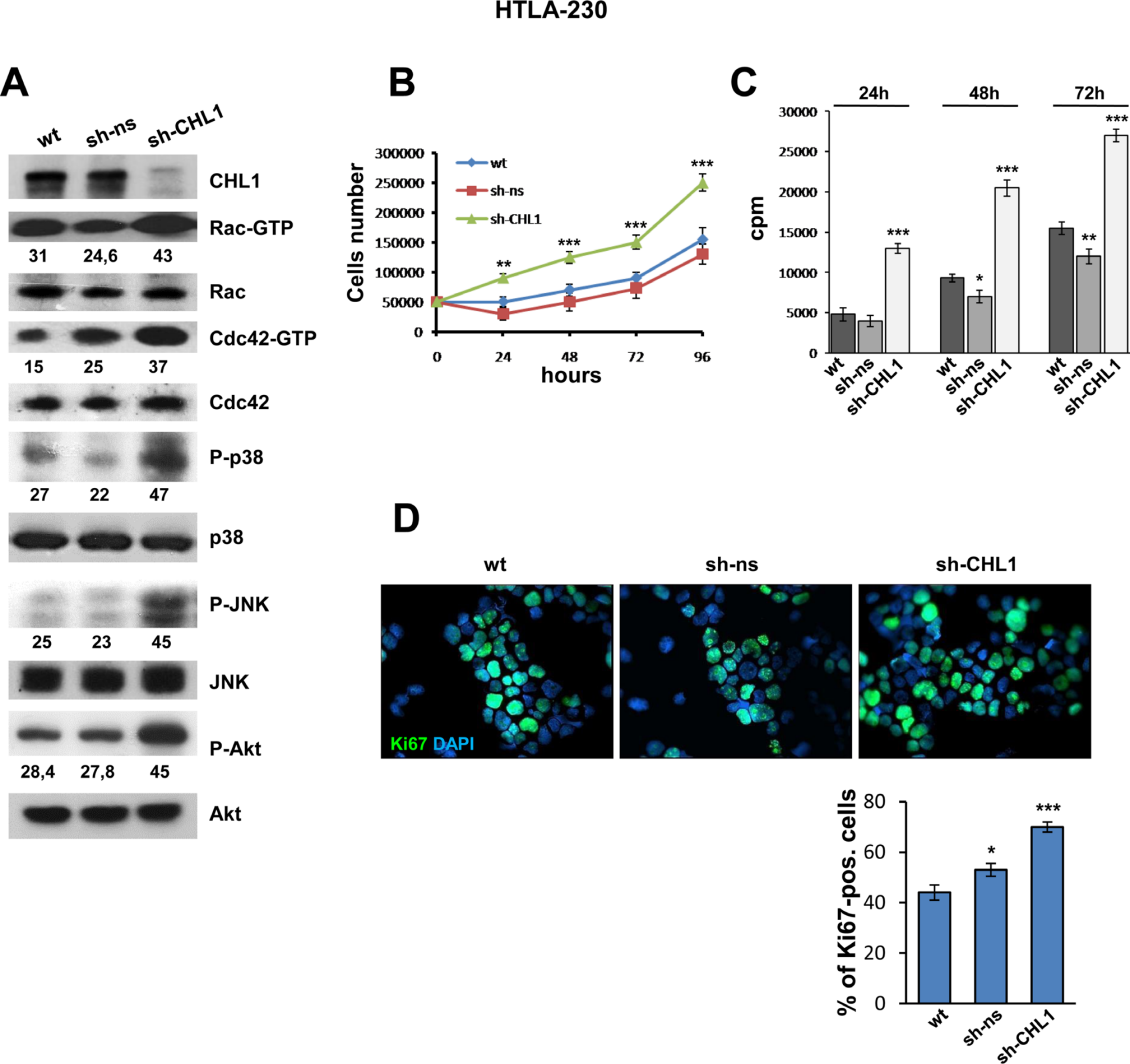
Down-regulation of CHL1 expression enhances growth of NB cells (**A**) Protein lysates from HTLA-230 cells wt, or transfected with sh-ns or with sh-CHL1, were subjected to GST-PAK pull-down assay and anti-Rac or anti-Cdc42 Western blot analysis. The same lysates were blotted again and probed with anti-phospho-p38, anti-phospho-JNK, and anti-phospho-Akt antibodies. Lower numbers indicate densitometric values normalized to each total protein expression. (**B**) HTLA-230 cells wt, or transfected with sh-ns or with sh-CHL1 were cultured for 4 days. Every day cells were trypsinized and counted (Three independent experiments ± S.D). (**C**) HTLA-230 cells wt, or transfected with sh-CHL1 or with sh-ns were subjected to the [3H]thymidine incorporation assay (cpm = counts per minute) (Three independent experiments ± S.D). (**D**) Immunofluorescence analysis of proliferating cells using anti-Ki67 (green). Cells were counterstained with DAPI to visualize nuclei (blue). (Magnification 40×). (Three independent experiments ± SD).

CHL1 silencing in HTLA-230 cells enhanced the activation of small GTPases, and namely approximately 1.5 fold for Rac, and 2 fold for Cdc42 in comparison with non-silenced or wt HTLA-230 cells (Figure 5A). Accordingly, p38 and JNK kinase phosphorylation was almost doubled in CHL1-silenced cells (Figure 5A). CHL1 silencing induced approximately 1.6 fold activation of pAkt too, a feature promoting cell growth and survival (Figure 5A). sh-CHL1 transfected cells proliferated approximately 2.9-fold more than controls (Figure 5B), and displayed significantly higher ^3^H-thymidine incorporation compared with HTLA-230 wt or transfected with sh-ns (Figure 5C). Finally, staining of HTLA-230 cells transfected with sh-CHL1 with anti-Ki67 mAb showed that Ki67 positive cells increased significantly (70%) in comparison with non-silenced control cells (53%) and wt cells (44%) (Figure 5D).

### *CHL1* inhibited migration, invasiveness and anchorage-independent colony formation of neuroblastoma cells

**Figure 6:**
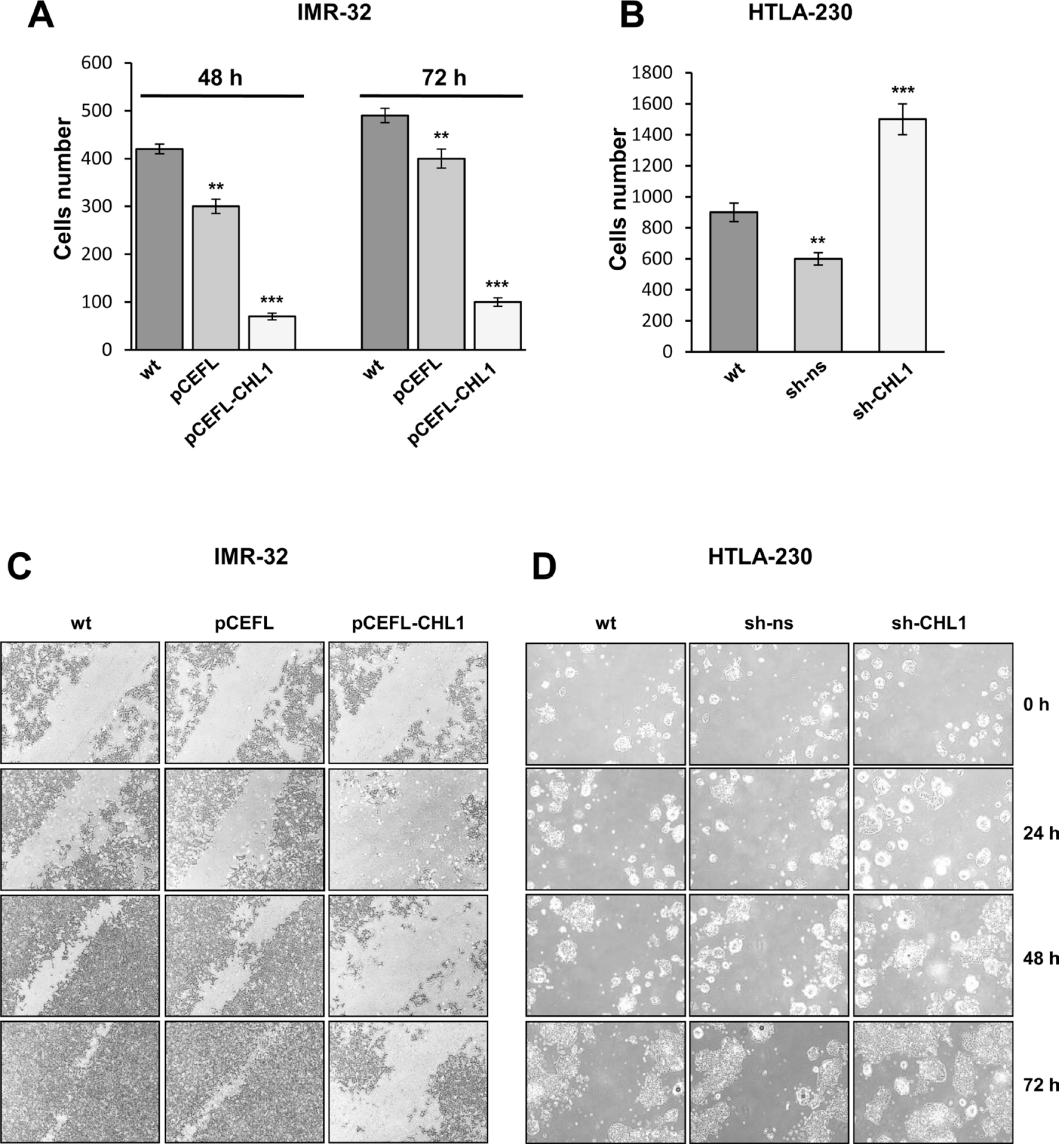
CHL1 expression influences NB cells migration and invasion (**A**–**B**) Migration assay for IMR-32 cells wt or transfected with pCEFL or with pCEFL-CHL1 (48 or 72 hours after transfection) (A) and for HTLA-230 cells wt or transfected with sh-ns or with sh-CHL1 (B). Cells were seeded on the upper chamber of a transwell insert and 24 hours later, migrated cells were detached from the lower side of the insert, collected and counted (Three independent experiments ± S.D). (**C**–**D**) Wound healing assay for IMR-32 cells wt or transfected with pCEFL or with pCEFL-CHL1 (C) and for HTLA-230 cells wt or transfected with sh-ns or with sh-CHL1 (D). Cells monolayers were scratched diagonally through the center of each well with a sterile tip (0 h) and photographed every 24 hours for 3 days under the light microscope (Magnification 4×).

**Figure 7:**
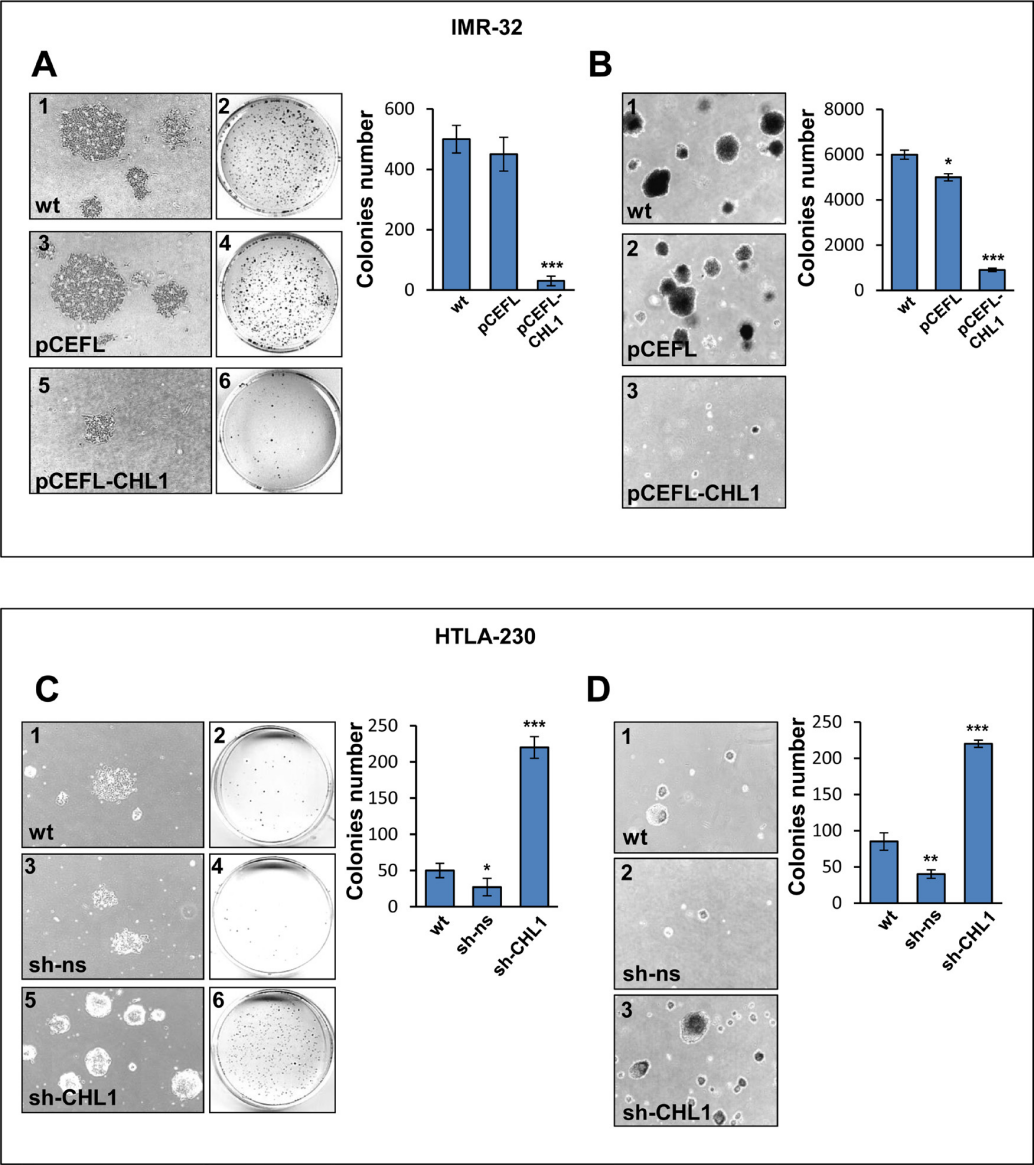
CHL1 expression affects colony formation and anchorage independent growth of NB cells (**A**) IMR-32 cells wt (1, 2), or transfected with pCEFL (3, 4), or with pCEFL-CHL1 (5, 6) were cultured for 15 days. The morphological characteristics of colonies were visualized by light microscope (1, 3, 5) (Magnification 4×), and the colonies were stained with crystal violet for quantification (2, 4, 6). (**B**) IMR-32 cells wt (1), or transfected with pCEFL (2) or with pCEFL-CHL1 (3) were cultured in soft agar for 20 days (Magnification 4×). (**C**) Colonies in HTLA-230 cells wt (1, 2), or transfected with sh-ns (3, 4), or with sh-CHL1 (5, 6) after 15 days culture. (**D**) Colonies in HTLA-230 cells wt (1) or transfected with sh-ns (2) or with sh-CHL1 (3) cultured in soft agar for 20 days. Histograms show mean colony numbers from three independent experiments ± S.D.

We next evaluated the effect of CHL1 expression on cell migration by a transwell assay, the ectopic expression of CHL1 resulting in five fold decrease of IMR-32 cell migratory response compared to controls (Figure 6A), while CHL1 down-regulation led to a migratory rates 2-fold higher than in HTLA-230 wt or transfected with sh-ns (Figure 6B). A significant inhibition of cell movement upon induction of CHL1 expression was detected in IMR-32 cells transfected with pCEFL-CHL1 respect to IMR-32 wt or transfected with pCEFL by wound healing assay (Figure 6C). These data indicated that CHL1 over-expression increased cell adhesion and decreased cell motility. Conversely, CHL1 silencing resulted in increased cell motion, HTLA-230 cells transfected with sh-CHL1 covering the higher percentage of empty space after 72 h respect to wt or control cells (Figure 6D). Colony forming assay showed a significant reduction in size and number of colonies formed by IMR-32 cells transfected with pCEFL-CHL1, compared with wt or control cells (Figure 7A). To evaluate whether CHL1 over-expression inhibited anchorage-independent colony formation, soft agar assays were performed. CHL1 over-expression inhibited anchorage-independent colony formation of NB cells compared to control cultures (Figure 7B). CHL1-silenced HTLA-230 grew much faster than the parental or control cells and gave an higher number of colonies both in normal cultures and in soft agar assay (Figure 7C, 7D). Thus, CHL1 knockdown evoked phenotypic changes opposite to those induced by CHL1 over-expression and indicators of a major tumorigenic potential.

### *CHL1* decreased neuroblastoma growth *in vivo*

**Figure 8:**
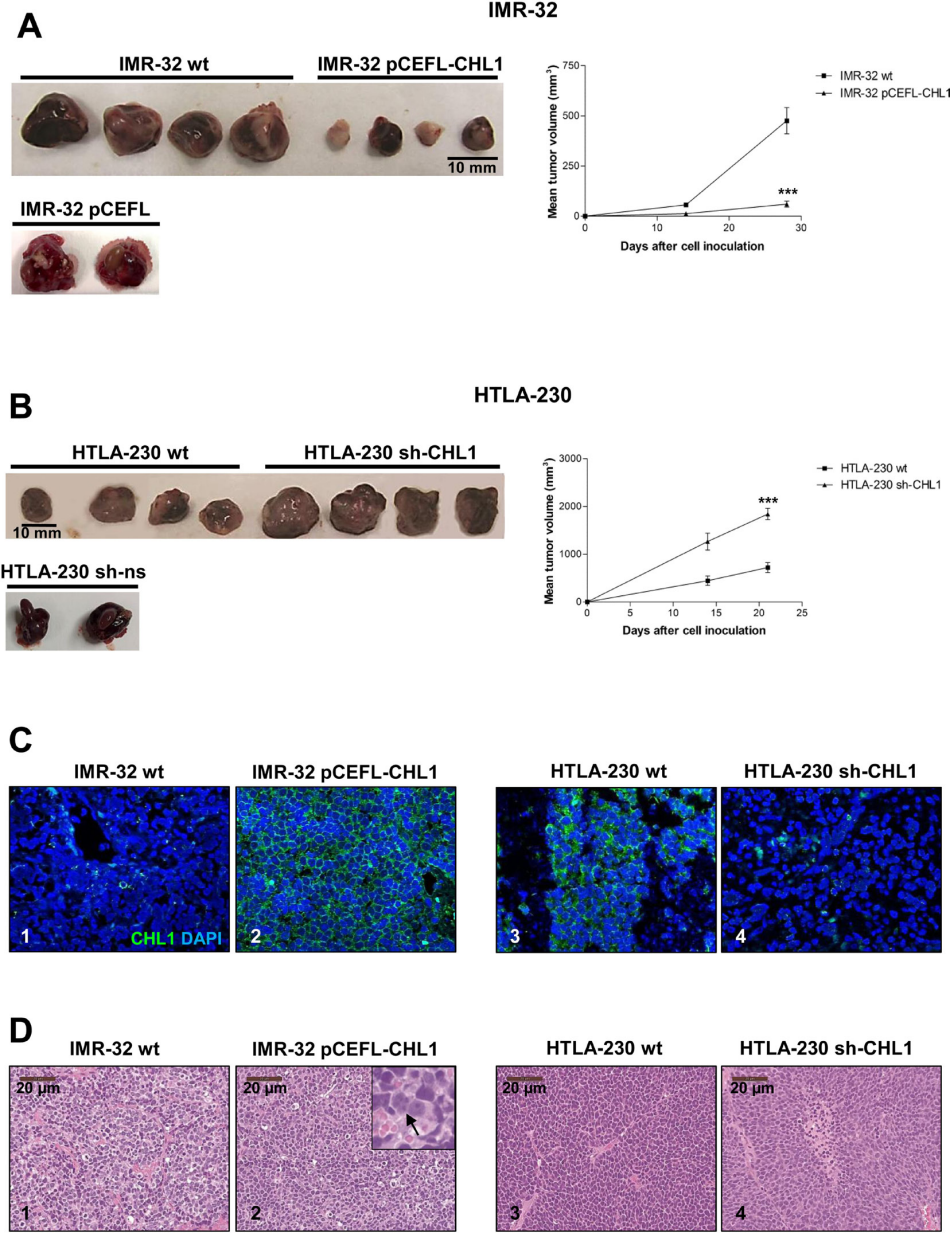
CHL1 decreases NB growth *in vivo* (**A**) Representative images of orthotopic NB tumors formed by IMR-32 cells wt, or transfected with pCEFL-CHL1, or with empty vector pCEFL, after 28 days from implantation. The average volume of tumors formed by IMR-32 cells transfected with pCEFL-CHL1 cells was smaller than that formed by control cells. (**B**) Representative images of orthotopic NB tumors formed by HTLA-230 cells wt, or transfected with sh-CHL1, or with empty vector sh-ns after 21 days from implantation. The average volume of tumors formed by HTLA-230 cells transfected with sh-CHL1 cells was larger than that formed by control cells. After 3 or 4 weeks, all mice were sacrificed and final tumor tissues were photographed. Tumor volumes were recorded with a caliper and were calculated according to the formula volume = π/6[ω_1_x(ω_2_)^2^]. The significance of differences between experimental groups and controls was determined by the unpaired *t*-test. (**C**) CHL1 protein located on the cell surface (green) as assessed by immunofluorescence with specific mAb in orthotopic tumors formed by IMR-32 cells wt (1) or transfected with pCEFL-CHL1 (2) or by HTLA-230 cells wt (3) or transfected with sh-CHL1 (4) (Magnification 40×). (**D**) Representative images showing the morphological appearance of the orthotopic tumors stained with hematoxylin/eosin. NB tumors developed by implantation of IMR-32 cells wt (1) or transfected with pCEFL-CHL1. The inset shows an enlargement of some differentiating cells (arrow) (2). NB tumors formed by implantation of HTLA-230 cells wt (3) or transfected with sh-CHL1 (4).

To assess *in vivo* tumorigenicity, IMR-32 cells wt or transfected with pCEFL-CHL1, and HTLA-230 cell wt or transfected with sh-CHL1 were implanted in the capsule of the left adrenal gland of nude mice. CHL1 over-expression significantly inhibited tumor growth compared with control mice. Thus, the average volume of tumors formed by IMR-32 cells transfected with pCEFL-CHL1 cells was significantly smaller than that formed by IMR-32 cells wt (*p* = 0.007 after 14 days and *p* = 0.0008 after 28 days) (Figure 8A). As shown in Figure 8B, tumors formed by CHL1 shRNA-transfected cells were significantly larger and grew faster than those formed by parental cells (*p* = 0.0066 after 14 days; *p* = 0.0004 after 21 days). These results indicate that CHL1 deficiency promotes the tumorigenicity of NB cells. We stained by immunofluorescence with anti-CHL1 mAb orthotopic NB tissue sections from IMR-32 cells wt or transfected with pCEFL-CHL1, and from HTLA-230 cell wt or transfected with sh-CHL1. The proportion of CHL1^+^ cells was significantly increased in tumors from IMR-32 cells transfected with pCEFL-CHL1 *vs* those formed by parental cells (93.5 ± 6% *vs* 2 ± 4%, *p* = 0.0001) (Figure 8C, 1 and 2). Conversely, CHL1^+^ cells were significantly decreased in tumors from HTLA-230 transfected with sh-CHL1 *vs* the tumors formed by parental cells (28 ± 8.2% *vs* 75 ± 2.7%, *p* = 0.0004) (Figure 8C, 3 and 4).

**Figure 9:**
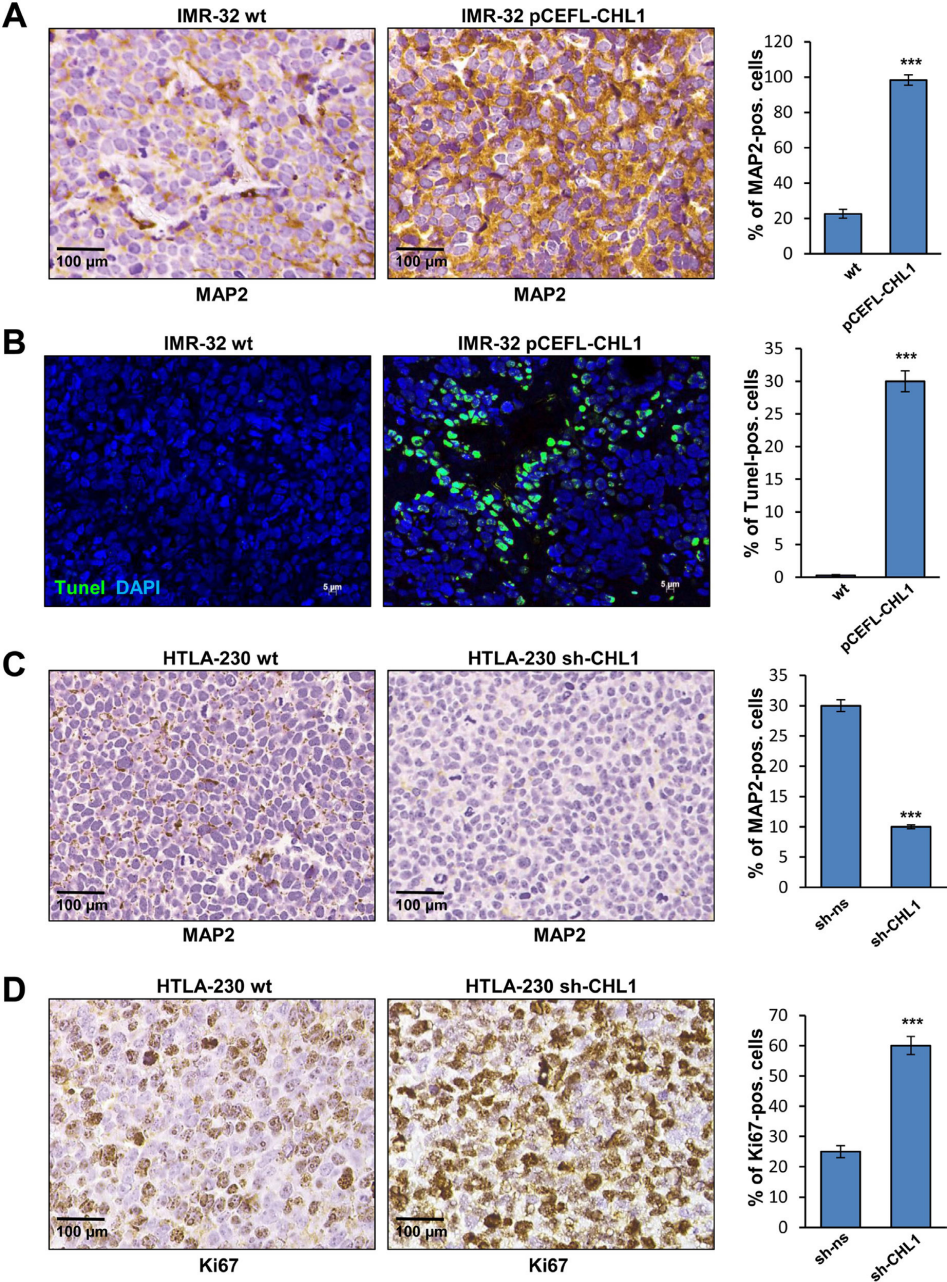
*In vivo* characterization of orthotopic NB (**A**) Immunohistochemistry analysis of MAP2 expression in orthotopic NB obtained by implantation of IMR-32 cells wt or transfected with pCEFL-CHL1. (**B**) Apoptotic cells in NB formed by implantation of IMR-32 cells wt or transfected with pCEFL-CHL1. TUNEL-positive apoptotic cells were detected by localized green fluorescence within cell nuclei counterstained with DAPI (blue) (Magnification 40×). (**C**) MAP2 immunohistochemical staining in NB obtained by implantation of HTLA-230 cells wt or transfected with sh-CHL1. (**D**) Ki67 immunohistochemical staining in NB formed by implantation of HTLA-230 cells wt or transfected with sh-CHL1. The histograms represent the percentage of MAP2^+^, Ki67^+^ and TUNEL^+^ cells.

**Figure 10:**
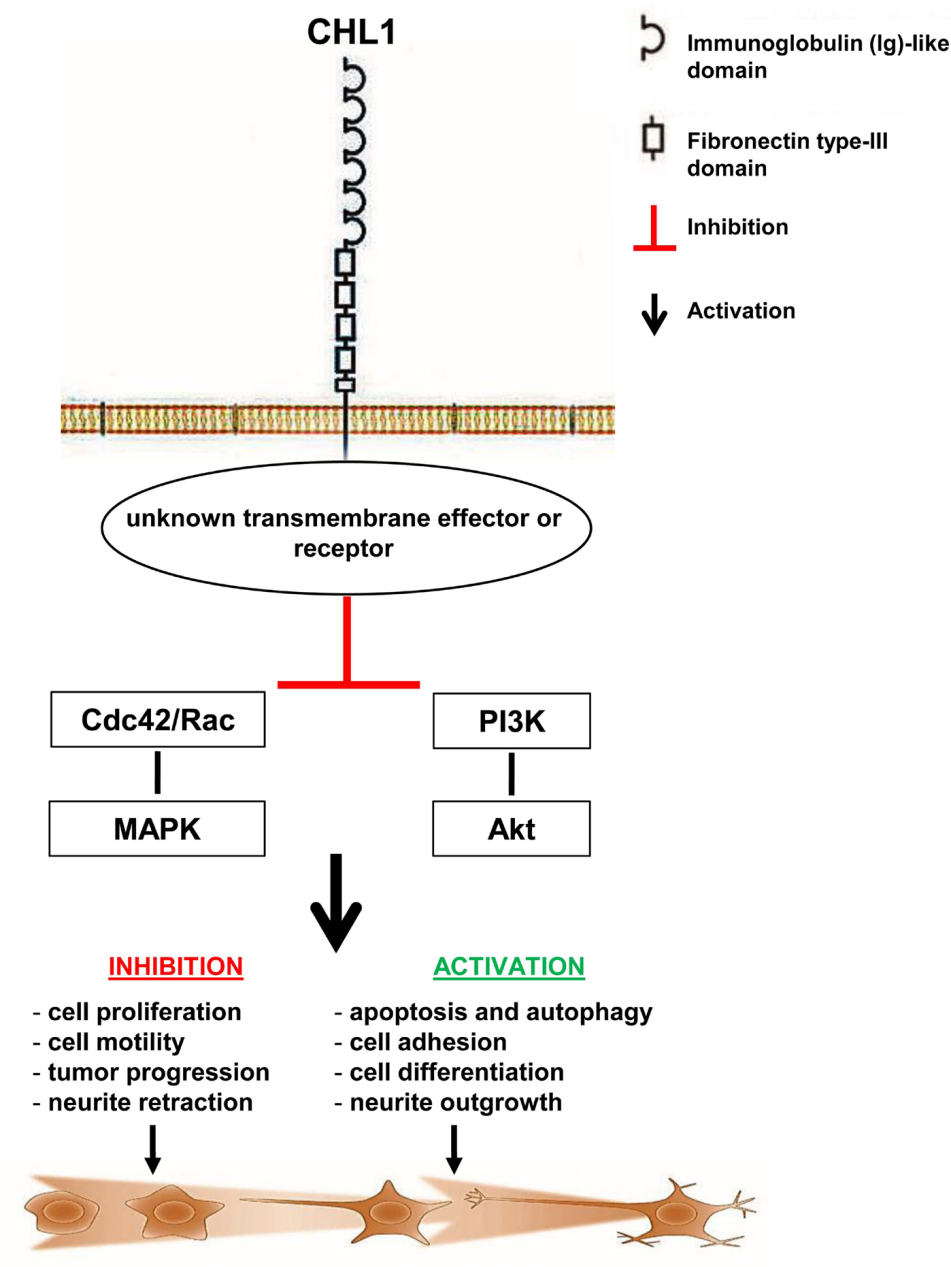
Proposed CHL1 signaling model In this model, CHL1 binds to yet unknown ligands and inhibits the activation of Rho GTPases, of related p38/JNK MAPK pathways, and of p-Akt. These events, in turn, result into enhanced tumor cell apoptosis and autophagy, differentiation, adhesion and neurite outgrowth, as well as into dampened cell proliferation and motility and tumor progression.

Histological examination of orthotopic tumors formed by IMR-32 cells transfected with pCEFL-CHL1 showed focal evidence of gangliar differentiation witnessed by scanty large bi-nucleated cells, not detected in control tumors (Figure 8D, 1 and 2). Conversely, histological features of orthotopic tumors formed by HTLA-230 cells transfected with sh-CHL1 were similar to those of control tumors (Figure 8D, 3 and 4). Interestingly, MAP2 expression, indicative of differentiated state, was significantly higher in tumors formed by IMR-32 cells transfected with pCEFL-CHL1 than in control tumors (Figure 9A). Tumors formed by IMR-32 cells wt displayed negligible proportions of apoptotic cells (0.1%), as assessed by TUNEL assay, whereas the CHL1-induced ones contained approximately 30% apoptotic cells (Figure 9B). Finally, a significant decrease of MAP2 expression in tumors from HTLA-230 cells transfected with sh-CHL1 highlighted the less differentiated status of these tumors (Figure 9C). Furthermore, staining with anti-Ki67 mAb showed a significant increase of proliferating Ki-67^+^ cells in tumors from cells transfected with sh-CHL1 *vs* control mice (Figure 9D). Consistent with our *in vitro* findings, these observations support the conclusion that CHL1 acts as a tumor suppressor in NB. Taken together, our results demonstrate that over-expression of CHL1 inhibits the activation of Rho GTPases, of related p38/JNK MAPK pathways, and of p-Akt inducing cell apoptosis and differentiation. Conversely, suppression of CHL1 promotes the activation of Rho GTPases, of related MAPK pathways, and of p-Akt inducing cell proliferation and tumor progression (Figure 10).

## DISCUSSION

In this study, we provided evidence of the tumor suppressive effect and prognostic potential of CHL1 in NB. Our results demonstrate the association of *CHL1* gene expression with NB patient outcome, including an association between low *CHL1* gene expression and the risk of relapse. Thus, *CHL1* expression predicts survival, outcome and disease status of NB patients, and restoration of *CHL1* expression or its associated pathways may represent a potential therapeutic strategy for NB. We have examined the expression of CHL1 in primary NB samples and found that CHL1 is lowly expressed in 76.4% of cases, and that 3p deletion induced a reduction of *CHL1* gene expression.

To investigate the impact of *CHL1* on NB growth we used two inducible cell models: i) the IMR-32 cell line, with low *CHL1* mRNA level and no detectable expression of CHL1 protein, and ii) the HTLA-230 cell line, with medium *CHL1* mRNA level and evident CHL1 protein expression. Both cell lines carry amplification of *MYCN* oncogene, which is associated with aggressive disease course and poor outcome [1, 2]. IMR-32 NB cells, which display undifferentiated morphology, were induced to differentiate into neuronal-like cells *in vitro* by over-expression of CHL1. Here, we found that over-expression of CHL1 is sufficient to induce significant apoptosis in NB. In parallel, CHL1 over-expressing IMR-32 cells underwent autophagy, evidenced by up-regulation of Beclin-1 and LC3-II, and acquired expression of the neural differentiation marker MAP-2. Concomitantly, over-expression of CHL1 inhibited activation of Rho GTPases, which regulate MAPK activation, and induced apoptosis in a fraction of IMR-32 cells by blocking the phosphorylation of JNK, p38, and Akt. The Rho family of GTPases represents a subgroup of the Ras superfamily of GTPases whose aberrant regulation has been associated with key features of aggressive tumor behavior [27]. Specifically, Rho GTPases play pivotal roles in the regulation of cell proliferation, survival, migration and cytoskeleton development [27]. Rho GTPases (such as Rac and Cdc42) regulate MAPKs activation in response to various stimuli [28]. Increased expression of Cdc42 was previously found to correlate with an undifferentiated NB phenotype [29].

P-Akt signaling regulates cell survival and growth, as well as glucose metabolism, cell motility, angiogenesis, and prevents apoptosis [30]. Furthermore, various oncoproteins and tumor suppressors interact with the Akt signaling pathway [30]. Apoptosis and autophagy, basic physiologic processes responsible for the maintenance of cellular homeostasis, are independent but related [31]. Accumulating evidence point to the essential roles for apoptosis and autophagy in the development and differentiation of the central nervous system [32]. Induction of autophagy promotes differentiation and neurite remodeling in the SH-SY5Y NB cell line [33], in early neural stem cells and progenitors [34], and in mouse NB cells (N2a cells) [35]. In this connection, activation of apoptosis and neuronal differentiation have been proposed to explain the phenomenon of spontaneous regression in NB [36]. In brief, over-expression of CHL1 in IMR-32 NB cell line abrogated anchorage-independent colony formation, inhibited proliferation and invasion, impaired migration and growth, induced neuronal differentiation, and dampened tumor growth in orthotopic xenografts mouse model. These findings demonstrate that CHL1 suppressed critical processes of malignancy in NB cells.

Knock-down of CHL1 in HTLA-230 NB cell line triggered colony formation and anchorage-independent growth, enhanced cell proliferation and migration, induced activation of Rho GTPases, decreased expression of MAP2 and of the autophagy protein markers Beclin1 and LC3 inducing neurite retraction, and accelerated growth in orthotopic xenografts mouse model.

Based upon our results, we propose that *CHL1* functions as a tumor suppressor gene in NB. Classical tumor suppressors genes have altered expression of both alleles resulting in loss of gene function. CHL1 maps to chromosome 3p26.3, a genomic region frequently involved in aberrations in NB [1]. The absence of bi-allelic loss of *CHL1* gene suggested that loss of a single allele of *CHL1*, as the result of 3p loss, may play a role in the pathogenesis of NB. Previous sequencing studies on primary NB have identified heterozygous loss-of-function mutations in *CHL1* gene [7]. We cannot exclude the possibility that one allele of *CHL1* is deleted and the other is mutated in a larger NB population. *CHL1* may function like haploinsufficient tumor-suppressor genes, where a partial loss of CHL1 may be sufficient to result in a cellular phenotype that leads to NB tumorigenesis. *CHL1* was listed as autosomal gene subjected to random monoallelic expression (RME) [37, 38]. RME is a process in which transcription occurs from only one of two homologous alleles in a diploid cell, independently of the genetic sequence and parental origin. The choice of which allele is expressed in a given cell is completely random, one would predict that human individuals heterozygous for a mutant allele at an affected locus will occasionally manifest a mutant phenotype. RME could play a role in NB pathogenesis by predisposing a proportion of cells to loss of function in the context of a mutation. The potential mechanisms regulating RME are involved in the control of gene expression and include epigenetic marks and non-coding microRNA (miR). In this respect, it has been shown that *CHL1* gene is a target of miR-21 in NB cells [39], even though the role of miR-21 in NB development and progression is still unclear. Similarly, miR-182 suppresses the expression of CHL1 mRNA through direct targeting of the 3′-untranslated region (3′-UTR) in papillary thyroid carcinoma [40]. Subsequently, it has been suggested that miR-590-5p acts as an oncogene by targeting *CHL1* gene and promotes cervical cancer proliferation [41]. MiR-10a, which targets CHL1, promotes cell growth, migration and invasion in human cervical cancer cells [42]. Recently, it has been reported that *CHL1* expression is shut-off by hypermethylation and that this epigenetic alteration is an independent prognostic factor in breast cancer [43] and colorectal cancer [44].

Taken together, our data identify a novel unbalanced developmental network in NB cells that may contribute to tumor aggressiveness. Characterization of the molecular pathways that lead to the development of NB may facilitate the identification of new targeted therapies. Improper alterations in downstream effectors of developmental apoptosis such as down-regulation of CHL1 may play a pathogenic role in NB by allowing neuronal progenitors to escape from developmental culling and thereby predisposing them to neoplastic transformation. A recent paper has demonstrated recurrent RAS/MAPK pathway mutations in NB after chemotherapy [45]. Previous studies have demonstrated that NB chemo-resistance was mediated by the PI3K/Akt pathway [46, 47]. Our findings provide support to the contention that CHL1 and its functions, such as p-Akt inhibition, may be targetable for therapeutic purpose in NB. Further studies are needed to unveil the fine mechanisms underlying CHL1-mediated tumor suppression.

## MATERIALS AND METHODS

### Patients

A total of 174 primary NB samples were enrolled on the basis of the availability of gene expression profile by Affymetrix GeneChip HG-U133plus2.0. Eighty-eight patients were collected by the Academic Medical Center (AMC; Amsterdam, Netherlands) [48, 49]; 21 patients were collected by the University Children's Hospital, Essen, Germany and were treated according to the German Neuroblastoma trials; 43 patients were collected at Hiroshima University Hospital or affiliated hospitals and were treated according to the Japanese NB protocols [50]; 22 patients were collected at Giannina Gaslini Institute, and treated according to Associazione Italiana Ematologia Oncologia Pediatrica (AIEOP) or International Society of Pediatric Oncology Europe NB (SIOPEN) protocols [51]. The data are stored in the R2 repository (http://r2.amc.nl) or in the BIT-NB Biobank of the Giannina Gaslini Institute. Informed consent was obtained in accordance with institutional policies in use. Tumor samples were obtained before treatment at the time of diagnosis. One hundred and ten patients had stage 1, 2, 3, 4 s disease, and 64 patients stage 4 according to the International Neuroblastoma Staging System [52]. Twenty-eight patients had *MYCN* amplification and 146 patients had *MYCN* single copy according to the copy number of the gene on chromosome 2. Age at diagnosis was defined as the patient's age before or after 1 year. Eighty patients aged less than 12 months, and 94 patients more than 12 month at diagnosis.

### Gene expression analysis

Gene expression profiles for the 174 NB tumors were obtained by microarray experiment using Affymetrix Gene-Chip HG-U133plus2.0 and the data were processed by MAS5.0 software according Affymetrix' s guideline [48].

### Cell culture

Human NB cell lines GI-ME-N, SH-EP-21N_mycn_on, LA-N-1, GI-LI-N, IMR-32, SH-EP-21N_mycn_off, SH-EP-2, SK-N-BE2(C), SK-N-SH, SK-N-F1 were obtained from ICLC-Interlab Cell Line Collection, San Martino-IST, Genova, Italy, HTLA-230 cell line was kindly provided by E. Bogenmann. The genomic identity of each line was regularly confirmed using array-CGH, and cell lines were routinely tested to confirm the lack of mycoplasma contamination.

### Real-time quantitative RT-PCR analysis

Total RNA from NB cell lines was extracted with TRIzol^®^ (Invitrogen, Waltham, MA, USA) and chloroform and controlled for integrity with an Agilent Bioanalyzer 2100 (Agilent Technologies, Santa Clara, CA, USA). RNA was quantified by NanoDrop (NanoDrop Technologies, Wilmington, DE, USA) and reverse-transcribed into doublestranded cDNA on a GeneAmp PCR System 2700 thermal cycler (Applied Biosystems, Waltham, MA, USA) using the SuperScript Double-Stranded cDNA synthesis kit (Invitrogen). qRT-PCR was performed on a 7500 Real Time PCR System (Applied Biosystems) in triplicate for each target transcript using SYBRGreen PCR Master Mix (Applied Biosystems) and 1.6 μM sense and antisense oligonucleotide primers (TIB Molbiol, Genova, Italy). The primers to detect human CHL1 were the forward 5′-GAA CTA TCC TTG CCA ATG CCA ATA T-3′ and the reverse 5′-TTC TGC CAG GAC ACG ACT GC-3′. qRT-PCR was performed in triplicate for each transcript and fluorescence was measured during the annealing step in each cycle. Quantitative gene expression data were normalized to the expression levels of the housekeeping control gene Glyceraldehyde 3-phosphate dehydrogenase (GAPDH). The specificity of the qRT-PCR products was proven by appropriate melting curves (specific melting temperature) and by the expected size of the PCR products, determined by gel electrophoresis.

### Plasmids, shRNAs and transfections

Full length human CHL1 gene was purchased from Sino Biological Inc. (Beijing, China) and sub-cloned into the eukaryotic expression vector pCEFL (kindly provided by J.S. Gutkind). IMR-32 cells were transiently transfected with the recombinant vector pCEFL-CHL1 and with the empty vector pCEFL as negative control. For generation of stable silenced CHL1 expression, HTLA-230 cells were transfected with: i) silencing CHL1 shRNA lentiviral plasmid (sh-CHL1) (a pool of three target-specific lentiviral vector plasmids by Santa Cruz Biotechnology, Dallas, TX, USA); ii) non-silencing control shRNA plasmid A (Santa Cruz Biotechnology). Stable transfected HTLA-230 cells were selected in DMEM supplemented with 0,4 μg/ml of puromycin (Sigma) for 14–21 days. Cells were transfected using Lipofectamine 2000 (Invitrogen, Waltham, MA, USA) according to the manufacturer's instructions. IMR-32 and HTLA-230 cell lines were chosen because they exhibit *MYCN* amplification, the hallmark of aggressive NB, and were reported having the ability to form tumors when injected orthotopically into adrenal gland [53, 54].

### Western blot analysis

Western blot analysis was performed as described previously [55]. Blots were probed with: monoclonal antibodies anti-MAP2 (Thermo Scientific, Waltham, MA, USA) and anti-PARP (Abcam, Cambridge, UK); polyclonal antibodies anti-CHL1 (Santa Cruz Biotechnology), anti-Beclin1 (Novus Biologicals, Littleton, CO, USA) and anti-LC3A (Cell Signaling Technology Inc., Danvers, MA, USA). Blots were reprobed with anti-β-Actin (Santa Cruz Biotechnology) as loading control. Bands signal intensity was measured by densitometry using Image Lab 6.0 software (ChemiDoc, Bio-Rad, Hercules, CA, USA) and normalized to loading control.

### *In vivo* GTPase activation assay

Whole-cell lysates from wt or transfected cells were collected and subjected to GST-PAK pull-down assay. The GST-PAK-CRIB domain fusion protein (residues 56–141) containing the Cdc42 and Rac binding region of human PAK1 was expressed and purified as described previously [55].

### Kinase activation assay

To assess the level of activated p38, JNK, and Akt, wt or transfected cells were starved 18 hours before lysis. Whole cell lysates were subjected to SDS-PAGE, transferred to PVDF membrane and the levels of activated p38, JNK and Akt were detected in Western blot by using phospho-specific monoclonal antibodies against P-p38 and P-JNK and polyclonal antibodies against P-Akt (Cell Signaling Technology). To detect the total amount of proteins loaded, the blot was re-probed with polyclonal antibodies against p38, JNK (Santa Cruz Biotechnology), and Akt (Cell Signaling Technology). Bands signal intensity was measured by densitometry using Image Lab 6.0 software (ChemiDoc, Bio-Rad) and normalized to each total protein expression.

### Cell growth and proliferation

To determine the growth rate, wt or transfected cells were plated in concentration of 5 × 10^4^ per well and cultured for 24, 48, 72 and 96 hours. Living cells were then detached and counted in Trypan blue. Cells proliferation was tested with the ^3^H-thymidine incorporation assay as described previously [56].

### Detection of apoptotic cells

Apoptosis was evaluated using *in situ* Cell Death Detection Kit (Roche, Basel, Switzerland) according to the manufacturer's protocol as described previously [53].

The following findings were considered to represent apoptosis: (i) marked condensation of chromatin; (ii) cytoplasmic fragments with or without condensed chromatin (apoptotic bodies); and (iii) intra- and extracellular chromatin fragments (micronuclei). Values represent percentages from at least 1000 counted apoptotic and non-apoptotic cells.

### Transwell migration assay and wound healing assay

Cell migration was assayed in Transwell chambers constituted of polycarbonate cell culture inserts with 8.0 μm pore size (Corning Costar, Cambridge, MA). Wt or transfected cells in concentration of 2 × 10^5^ were suspended in 100 μl of serum-free medium and added to the upper chamber. Inserts were incubated for 24 hours at 37°C with 600 μl of complete medium in the lower chamber. Cells transmigrated on the underside of the insert were detached using 5 mM EDTA and a plastic scraper, collected together with cells eventually fallen in the underlying well and counted. Not migrated cells were collected from the upper chambers and stained with Trypan Blue to verify their viability. To test the cell invasion capability, wt or transfected cells were plated and grown to ~70% of confluence, then the mono-layers cells were scratched vertically down the center of each well with a sterile tip and each well was washed with fresh medium. Cells were cultured for three days and the area covered by the cells after 72 hours was measured and compared with the wound scratched at time 0.

### Colony formation assay and anchorage-independent growth assay

Wt or transfected cells were plated (2 × 10^4^ cells per plate) and cultured for 15 days. Colonies were then fixed in 4% paraformaldehyde, stained with crystal violet (0,1% in 20% methanol) and scored.

Colonies were photographed under the CKX41 phase-contrast microscope Olympus (Tokyo, Japan) equipped with the Altra-20 digital camera, and with the AnalySIS^®^ getIT imaging acquisition software (Olympus). For soft agar assay, wt or transfected cells were suspended in DMEM medium with 10% FBS and 0.3% agarose (SeaKem^®^ ME by Lonza, Basel, Switzerland), and plated on top of a 0.6% agarose layer at the concentration of 2 × 10^4^ cells per p60 plate. Growing colonies were scored after 20 days and photographed under phase-contrast microscope.

### Immunohistochemistry analysis

Paraffin-embedded sections (4 μm) were deparaffinized in three xylene washes followed by a graded alcohol series, subjected to antigen retrieval performed with 10 mM sodium citrate buffer, and then blocked with BSA solution for 1 h at RT. They were incubated with primary antibodies against MAP2 (Thermo Scientific) and Ki67 (Abcam) overnight at 4°C, washed with PBS, incubated with secondary antibodies for 30 min at RT, and developed with DAB reagent. All sections were counterstained with hematoxylin, and then dehydrated with ethanol and xylene. Cover-slips were mounted and slides observed under light microscopy.

### Immunofluorescence analysis

Indirect immunofluorescence was performed on 4-μm-thick formalin-fixed, paraffin-embedded tissue sections or on cytospins as previously described [53]. The following antibodies were used: anti-Ki67 (diluted 1:100, Abcam) and anti-CHL1 (diluted 1:50, Abcam). Antigen retrieval slides were incubated with primary antibodies overnight at 4°C. Secondary antibodies were goat anti-mouse IgG conjugated to Alexa-488 or goat anti-rabbit IgG Alexa- Green (1:200; Invitrogen, Germany). After washing, the slides were counterstained with 4′,6′-diamidino-2-phenylindole (DAPI) (Vector Laboratories, Peterborough, United Kingdom) and cover-slipped. Isotype-matched non-binding mAbs were used in all antibody-staining experiments to exclude non-specific reactivity. The slides stained by immunofluorescence were examined at low magnifications (40×), and the positive cells were counted using fluorescence microscopy (Axio Imager M2 equipped with ApoTome System, Carl Zeiss, Oberkochen, Germany). The Ki67 and CHL1 scores were calculated as the percentage of positive cells.

### Animal experiments

All procedures involving animals were performed in respect of the National and International current regulations (D.lgs 26/2014 art.23; European Union Directive 2010/63/EU). Moreover, our experimental protocols were approved by the Italian Institutional Animal Care (N°1033/2016-PR). Female Hsd athymic nude (*nude/nude*) mice, 5 weeks old, were purchased from Envigo (Huntingdon, UK). Thirty-two mice (*n* = 8 mice per group) were anesthetized with ketamine (Imalgene 1000, Merial Italia SpA., Milan, Italy), subjected to laparotomy and injected with IMR-32 cells wt or transfected with pCEFL-CHL1, and with HTLA-230 cells wt or transfected with sh-CHL1 (1 × 10^6^ cells in 10 μl of saline solution) in the capsule of the left adrenal gland as previously described [53]. Two mice per group were injected with cells transfected with the empty vectors to verify that there was no difference with wt cells effect. No mice died as a result of this treatment. Mice were sacrificed when signs of poor health became evident. The orthotopic tumors were harvested after 2 or 3 weeks if derived from HTLA-230, and after 2 or 4 weeks if derived from IMR-32. Tumor volumes were examined, measured and recorded, after which tumors were formalin fixed and paraffin sections analyzed by haematoxylin-eosin staining.

### Statistical analysis

Kaplan-Meier analyses and comparison of *CHL1* expression between different patient subgroups for the two NB cohorts were performed online in the R2 platform (http://r2.amc.nl). Mann-Whitney *U* test was used for the comparison of *CHL1* expression between different patient subgroups. The Elbow method was used to find the appropriate number of clusters in a dataset. For all analyses, the significance of differences between experimental samples and controls was determined by the ANOVA analysis with Bonferroni's Multiple Comparison test: ^*^p < 0.05, ^**^p < 0.01, ^***^p < 0.001.

## Abbreviations

NB: neuroblastoma
CHL1: cell adhesion molecule close homolog of L1
wt: wild type
MAP-2: Microtubule Associated Protein-2
PAKs: p21-activated kinases
MAPKs: mitogen-activated protein kinases
p38: p38 mitogen-activated protein kinase
JNK: Jun N-terminal kinase
Akt: RAC-alpha serine-threonine-protein kinase
PI3K: phosphoinositide 3-kinase
shRNA: small hairpin RNA
sh-ns: non-silencing control shRNA
RME: random monoallelic expression
miR: microRNA
3′-UTR: 3′-untranslated region.
